# Effect of Heat Treatment on the Dry Sliding Wear Behavior of the Mg-3Zn-0.4Ca Alloy for Biodegradable Implants

**DOI:** 10.3390/ma16020661

**Published:** 2023-01-10

**Authors:** Nuria Pulido-González, Sonia García-Rodríguez, Belén Torres, Joaquin Rams

**Affiliations:** Departamento de Matemática Aplicada, Ciencia e Ingeniería de Materiales y Tecnología Electrónica, ESCET, Universidad Rey Juan Carlos, C/Tulipán s/n, Móstoles, 28933 Madrid, Spain

**Keywords:** magnesium alloys, dry sliding wear, heat treatment, precipitation, biodegradable implants, Mg-Zn-Ca alloys

## Abstract

The wear behavior of the Mg-3wt.% Zn-0.4wt.% Ca (ZX30) alloy was tested using a pin-on-disc configuration with AZ31 alloy discs as counterparts under dry sliding conditions. The ZX30 alloy was tested in different states: as-cast, solution-treated, peak-aged, and over-aged. Wear rates and friction coefficients were measured at different loads and sliding speeds. Abrasion and oxidation were the main wear mechanisms found in all the conditions tested. Moreover, aluminum oxides were detected on the worn surfaces, which indicates the presence of an adhesive wear mechanism. The wear behavior of the studied ZX30 alloy showed a greater tendency towards oxidative wear than other Mg alloys, and the microstructure observed strongly affected the wear behavior.

## 1. Introduction

Magnesium alloys are key materials in the evolution of many industries [1], and they have also emerged as potential biodegradable implant materials because of their low density (1.75–1.85 g/cm^3^) and their high specific mechanical properties making them a suitable substitute for other denser materials. Mg-based alloys reduce the stress-shielding phenomenon, which occurs when an implant is much more rigid than the bone and limits its healing. Another significant advantage of Mg alloys is their biodegradability when immersed in a body fluid. The dissolution of an Mg implant causes the incorporation of all its alloying elements into the body tissues. Therefore, it is preferable to use alloying elements that appear naturally in the body, such as Ca and Zn, which are considered safe for biomedical applications [2]. Ca improves the mechanical properties of Mg alloys and their corrosion resistance in multiple environments [3,4], while Zn improves strength and castability when combined with Ca, Si, or Mn [5].

On the other hand, Mg alloys are heat-treatable, and different surface treatments have been applied to these alloys, from the conventional treatment in furnaces [6] to laser processing ones [7]. C. J. Bettles et al. [8] and F. Czerwinski et al. [9] reported that small additions of Ca in Mg-Zn alloys can enhance the age-hardening response by the refinement of the microstructure after precipitation. Heat treatments produce different grain sizes and volume fractions of secondary phases in Mg-based alloys, which significantly influence their corrosion behavior [2]. Some prior studies have shown that the application of heat treatments can modify the hardness and the microstructure of Mg-Zn-Ca alloys [10], and that this induces a relevant change in the corrosion mechanisms of these alloys.

Despite the promising characteristics of Mg alloys, they present a poor wear response, as has been studied in many such alloys used as structural materials [11]. Considerable efforts have been devoted to understanding the wear behavior of Mg-based alloys and to improving their performance. Nevertheless, many of these studies have been conducted using a harder material as the counterbody, not simulating a real situation developed in an implanted prosthesis. There is little knowledge of the wear behavior of the Mg-Ca-Zn alloys, and the effect of heat treatments has not been established. Some previous results regarding these alloys show that the incorporation of high Zn content reduces the wear because of the reduced grain size and increased hardness [12]. Other studies have evaluated the microstructural change caused by heat treatments and its effect on these alloys’ corrosion behavior [10], but there are many aspects in which the role played by the composition and the manufacturing conditions in the wear behavior have not been differentiated.

The present work studies the effect of the resulting microstructure after heat treatment on the wear behavior of the ZX30 alloy under dry sliding conditions using the pin-on-disc test using an Mg alloy as the counterbody. The effect of the process variables (sliding speed and applied load) on the wear rate and wear mechanisms have been established considering the microstructure obtained in each studied condition.

## 2. Materials and Methods

### 2.1. Materials

Mg-3Zn-0.4Ca (hereafter ZX30) magnesium alloy plates were supplied by Helmholtz Zentrum Geesthacht (Germany) with the nominal composition shown in Table 1. The alloy was cast by permanent mold indirect chill casting in Ar—0.2% SF_6_ atmosphere at 720 °C. Then, Ca and Zn were added, stirred for 5 min, and cast into a mold. The details of the casting process can be found elsewhere [13].

### 2.2. Heat Treatments

Samples of the ZX30 alloy were solution-treated at 450 °C for 24 h in an electric furnace and then quenched in water. The aging treatment was carried out at 180 °C in an oil bath for up to 24 h, as reported previously in a work in which the evolution of hardness with aging time was established [10]. The Vickers microhardness test with an applied load of 0.1 kg was used to measure the microhardness of the samples from 10 individual indentations on each specimen. Table 2 shows the aging times used and the Vickers hardness values measured under the different studied conditions. Samples are referred to as as-cast, solution-treated, and two heat-treated categories: peak-aged (aged for 4 h) and over-aged (aged for 24 h).

### 2.3. Wear Tests

Wear tests were carried out under dry sliding conditions at room temperature in a pin-on-disc tribometer (Microtest MT/30) that records the friction coefficient and wear depth with the sliding distance. Two different loads and sliding velocities were used: 2 N and 20 N and 0.05 m s^−1^ and 0.1 m s^−1^, respectively. An optimized sliding distance of 200 m was established in prior tests. ZX30 samples were used in a pin configuration with the following dimensions: 15 mm × 2.5 mm × 2.5 mm. All the samples were ground with different emery papers of up to 1200 grit to homogenize the exposed areas before testing.

An AZ31 Mg alloy, the composition of which is shown in Table 3, was used as the counterbody. It was supplied by Luxfer Mel Technologies (UK) in the form of an extruded, rolled sheet with the following dimensions: 250 × 250 × 3 mm. Most studies use a steel counterbody, but a couple that would involve an Mg alloy and a steel is not likely in biomedical applications. The AZ31 Mg alloy contains aluminum, which allows differentiating between the material coming from the pin and that from the disc by the presence of this element in this study.

Both materials were cleaned before the wear tests using isopropanol to avoid the presence of humidity and non-desirable films such as grease. Three tests were conducted for each wear condition studied. Samples were weighed before and after the wear tests to determine the mass loss during the test. Volume loss and wear rate were determined using the alloy density.

The wear behavior was determined using Archard’s law (Equation (1)) [14]:

(1) $$\frac{V}{L}=K\frac{W}{H}=kW$$

where *V* is the wear volume, *L* is the sliding distance, and the coefficient *V*/*L* identifies the wear rate as the volume lost in the wear track per unit of sliding distance. In the case of the application of Archard’s law, the wear rate is proportional to the applied load (*W*), and inversely proportional to the hardness (*H*), with *K* being Archard´s constant and *k* the specific wear rate.

### 2.4. Microstructural and Compositional Characterization

A scanning electron microscope (SEM, Hitachi S-3400 N) was used for the microstructural characterization, and an energy-dispersive X-ray spectrometer (EDS, Bruker AXS Xflash Detector 5010) was used to identify the composition of the worn surfaces and the debris. The phases in the alloy were identified by an X-ray spectrometer (D8 AD-VANCE Bruker) using monochromatic KαCu (1.54056 A) as the radiation source, operating at a voltage of 40 kV and a current of 40 mA. The obtained patterns were indexed by using the PFD-4 + 2019 software, which contains the ICDD (International Centre for Diffraction Data).

## 3. Results and Discussion

### 3.1. Microhardness and Microstructure

**Figure 1:**
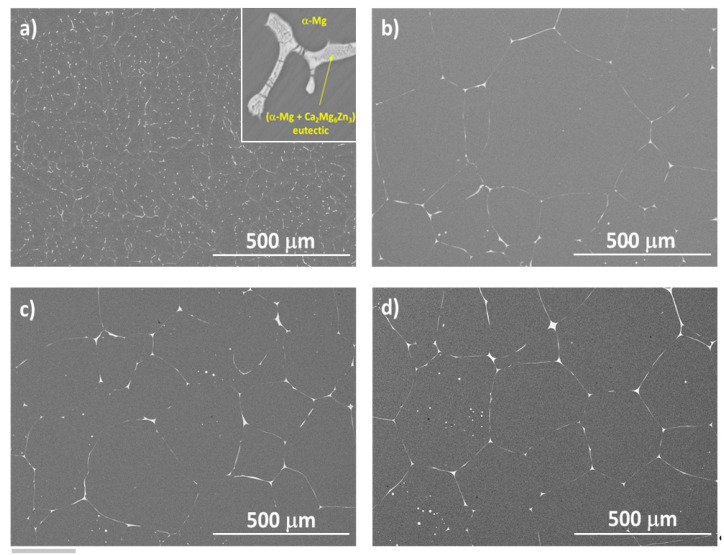
SEM micrographs of the ZX30 alloy samples: (**a**) as-cast, (**b**) solution-treated, (**c**) peak-aged, and (**d**) over-aged.

Figure 1 shows SEM micrographs of the ZX30 alloy in the different conditions tested. The as-cast alloy consists of α-Mg grains and large α-Mg + Ca_2_Mg_6_Zn_3_ eutectic phases located preferentially at the GBs (Figure 1a). After solution and aging treatments, the ZX30 alloy exhibits coarser and more equiaxed grains with a more continuous precipitates configuration at the GBs, and spherical particles within the grains are also observed (Figure 1b–d).

According to the microhardness evolution with aging time (Table 2), the as-cast and solution-treated specimens showed similar hardness values of ~61 HV and ~63 HV, respectively. After 4 h of isothermal aging at 180 °C, the alloy reached its peak hardness with a maximum of ~74 HV. Finally, after 24 h, the hardness declined to ~64 HV.

**Figure 2:**
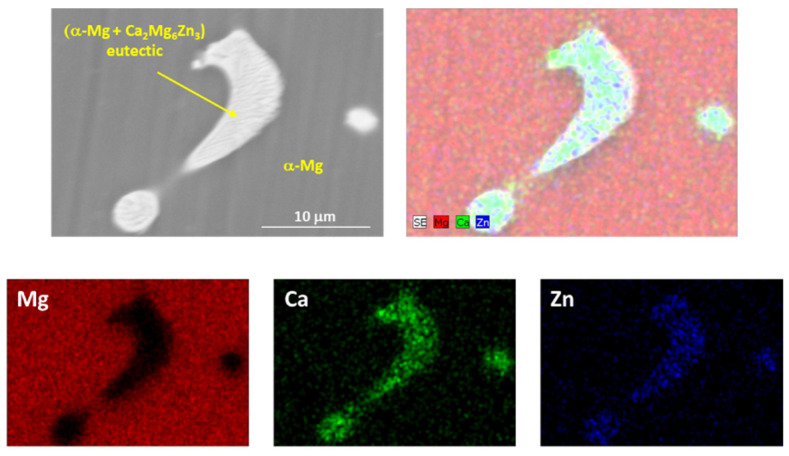
Detail of an α-Mg + Ca_2_Mg_6_Zn_3_ eutectic precipitated in the alloy.

The evolution of hardness has been associated with the evolution of the microstructure. In the as-cast alloy, the distribution of α-Mg grains and large α-Mg + Ca_2_Mg_6_Zn_3_ eutectic phases were located preferentially at the GBs of the alloy, but the distribution was associated with uncontrolled cooling of the alloy, so it was not homogeneous. Figure 2 shows an EDS mapping analysis of an α-Mg + Ca_2_Mg_6_Zn_3_ eutectic precipitated in the alloy.

After solution and aging treatments, Zn-rich precipitates about 200–500 nm in size were found together with fine Ca_2_Mg_6_Zn_3_ particles [6], but the hardening effect seems to be limited in this alloy due to the reduced number of particles dissolved during solution treatment. In the heat treatments that lasted longer than four hours, the precipitates do not further distribute in the alloy but engross the continuous phase at the GB.

Figure 1b,c show that after the heat treatments, the GB precipitates show a more connected configuration, and the heat-treated samples show coarser and more equiaxed grains compared to the as-cast state. As the precipitates’ dissolution is scarce in this alloy, the differences in volume fraction and precipitate size among the different heat-treated samples are not very noticeable [8]. However, the precipitation of Zn-rich precipitates and fine Ca_2_Mg_6_Zn_3_ particles is higher in the peak-aged condition, producing higher hardness values for this alloy.

Although it could be expected in these systems to find precipitation of the Mg_2_Ca phase, it was observed by Hofstetter et al. [15] that this has not been observed either by EDS, DRX, or transmission electron microscopy (TEM). This circumstance has been also considered by other authors such as Zhang et al. [16], Bakhesheshi et al. [17], and Lu et al. [2], who have also indicated that in Mg-Zn-Ca alloys with high Zn/Ca atomic ratios (>1.2, approximately), this phase is not observed.

### 3.2. Wear Rate and Friction Coefficient

**Figure 3:**
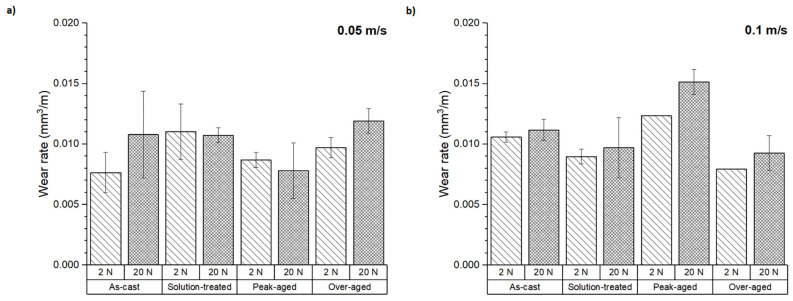
Wear rate of the ZX30 alloy applying 2 N and 20 N loads at sliding speeds of (**a**) 0.05 m s^−1^ and (**b**) 0.1 m s^−1^.

Figure 3 shows the volumetric wear rates of the ZX30 alloy in different conditions after a sliding distance of 200 m. In this alloy, the as-cast and the peak-aged samples showed the best behaviors at the lowest sliding speed. However, the volumetric wear rate of the peak-aged sample increased at 0.1 m s^−1^. At this sliding speed, the wear rate increases with the applied load, being lower for the solution-treated and over-aged conditions.

ZX30 alloy wear rates are mainly dependent on the applied load, being higher at 20 N. This observation was also made by D. Mehta et al. [18], who noted that the wear rates of two different Mg alloys, AZ91D and AS21, were more dependent on the applied load than on the sliding speed. They concluded that a higher load being applied led to higher wear rates. J. An et al. [19] also established that higher loads lead to superior wear rates, although the presence of some intermetallic phases with high thermal stability in the alloy could improve its wear behavior.

There is some controversy regarding the role of the different phases of the alloy in the wear behavior. On the one hand, D. Wan et al. [20] observed a decrease in the wear resistance of an Mg-Zn-Y alloy as the isothermal treatment time increased due to the dissolution and refinement of the I-phase. The I-phase is harder than the matrix, so it can restrain some wear mechanisms. On the other hand, after the application of heat treatment to the Mg-11Y-5Gd-2Zn alloy, M. Hu et al. [21] observed an increase of the Mg_12_Y_1_Zn_1_ phase, which appeared to be distributed randomly in the matrix. This phase has higher strength and better thermal stability than the a-Mg matrix, so it can hinder the material flow during wear testing, decreasing the wear rate of the alloy. A similar result was reached by Y. Hong et al. [22]. They determined that the T6 treatment applied to an extruded AZ91 alloy with 2.1 wt.% Y led to better wear resistance because of the distribution of large amounts of fine Mg_17_Al_12_ particles within the grains, which improved the alloy’s microhardness, and hence its wear behavior. As shown in Figure 3, both behaviors can be observed in this study. At the lower sliding speed, the peak-aged condition presented lower wear rates compared to the other conditions. After solution treatment, some α-Mg + Ca_2_Mg_6_Zn_3_ eutectic phases dissolved and precipitated as fine particles within the grains with higher hardness and thermal stability than the matrix, which could improve the wear resistance of the alloy. However, at 0.1 m s^−1^, the trend changes. The numerous and large secondary phases at the GBs could act as barriers against the material flow during the test. However, at the peak-aged condition, the dissolution of some α-Mg + Ca_2_Mg_6_Zn_3_ eutectic phases and their subsequent precipitation as fine particles within the grains led to a decrease in the wear resistance due to the decrease of coarser particles located at the GBs. This behavior can be also analyzed because of the greater friction coefficient at this sliding speed, as it would have caused a rise in the contact temperature and increased the tendency of the magnesium sample toward corrosion and plastic deformation. The presence of new mechanisms could explain the evolution observed in the specific wear rate.

Furthermore, H. Q. Sun et al. [23] determined that the grain refinement strengthening effect improved the wear resistance when they studied the wear behavior of the nanocrystalline layers generated by surface mechanical attrition treatment (SMAT) on an AZ91D alloy. F. Mert [24] also concluded that the mechanical strength was directly related to the grain size, similar to the hardness according to the Hall–Petch equation. For this reason, the refinement of the grains led to higher hardness, thus improving the wear resistance.

**Figure 4:**
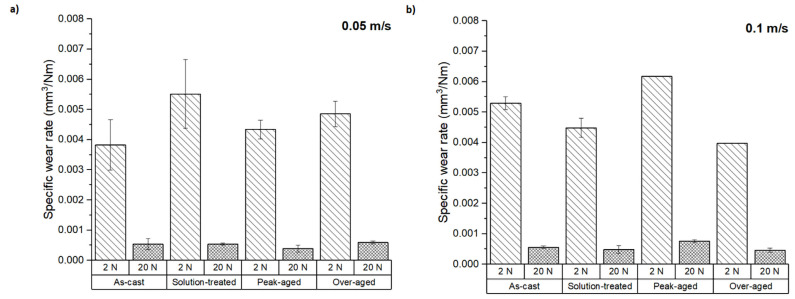
Specific wear rates at sliding speeds of (**a**) 0.05 m s^−1^ and (**b**) 0.1 m s^−1^.

Figure 4 shows the specific wear rates of the tested samples as defined by Archard’s law (Equation (1)). In all conditions, the specific wear rate was strongly dependent on the applied load. At 20 N, the specific wear rate was lower at all the conditions studied, irrespective of the sliding speed used. The specific wear rate showed values lower than 0.007 mm^3^ N^−1^ m^−1^ at 2 N and values below 0.001 mm^3^ N^−1^ m^−1^ at 20 N. These results suggest that different wear mechanisms could have acted at the loads tested. At 2 N load, the as-cast and peak-aged samples at low speed and the solution-treated and over-aged conditions at high-speed showed the lowest specific wear rate values. These results are consistent with those observed in Figure 3.

The specific wear rate, *k*, is a useful indicator of the wear behavior of a material when the interaction between the pin and the counterbody is dominated by the actual contact area. This usually happens when a soft material is tested against a much harder one, and when abrasion and adhesion are the predominating wear mechanisms. In the system studied, the hardness of the pin and counterbody are similar; this may be conducive to a condition in which there would be a transition region between the wear of the pin and that of the counterbody that makes it very sensitive to the presence of debris that would cause a three-body abrasion mechanism [25]. The presence of instabilities and a strong dependence on the load were also observed in the abrasion of tool steels [26]. Similarly, the changes observed in the specific wear rate under the different testing loads prove the presence of different wear mechanisms, such as oxidation or plastic deformation. Also, the evolution of the specific wear coefficients with heat treatment indicates the variation of the wear mechanisms in each studied condition.

**Figure 5:**
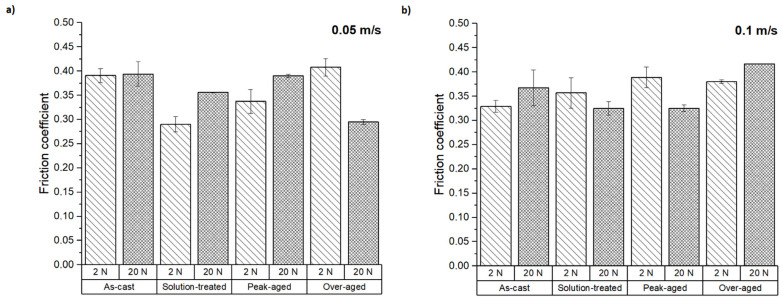
Friction coefficients at sliding speeds of (**a**) 0.05 m s^−1^ and (**b**) 0.1 m s^−1^.

The friction coefficients obtained for the ZX30 alloy under different wear test conditions are shown in Figure 5. For about 50 m of sliding distance, an initially unstable friction coefficient was observable in all conditions tested. Exceeding this value, the friction coefficient value became quite constant, showing a value between 0.30–0.40 for all the tested samples under the different test conditions.

The measured friction coefficients were high compared to other systems in which an Mg alloy is tested against a counterbody made of hardened steel, which produced friction coefficients in the 0.05–0.14 range [27]. The friction values observed on the contact between a rolled AZ31B alloy and the 6061-T6 aluminum alloy were 0.10–0.16 depending on the load and relative orientation of the rolling direction and the wear track [28], and the value increased with the applied load. The hardness values of the Mg and Al alloys used were ~62 and ~110 HV, respectively, so the difference between them was still much greater than those of the materials tested in this work. S. D. Wang et al. [29] observed a decrease in the friction coefficient of a heat-treated condition compared to the as-cast Mg-Zn-Y-Zr alloy under dry sliding conditions. In this study, there was not a clear relationship between the load and the sliding speed used. This indicates that friction is not governed by abrasion mechanisms and that other wear mechanisms such as adhesion could be promoting the increase of the friction coefficient, while others such as oxidation wear could be reducing it.

### 3.3. Determination of the Surface Elementary Composition

**Figure 6:**
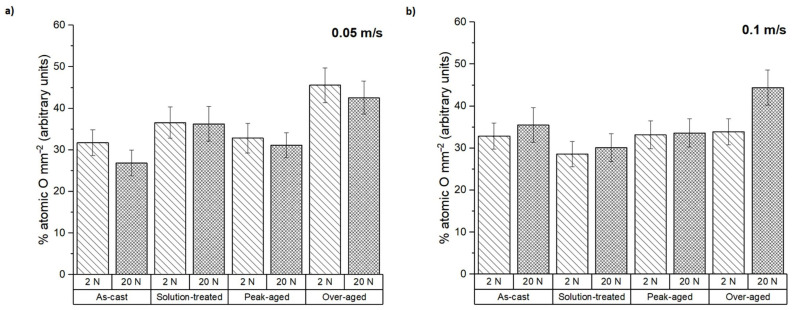
The atomic percentage of oxygen on the worn surface at sliding speeds of (**a**) 0.05 m s^−1^ and (**b**) 0.1 m s^−1^.

The average composition of the pin surfaces was determined by EDS to complement the analysis of the wear mechanisms. The atomic percentage of oxygen present on the surfaces of the samples after testing is represented in Figure 6. It was mainly independent of the test conditions, especially at the highest sliding speed. The over-aged condition showed the highest oxygen proportion in all cases.

**Figure 7:**
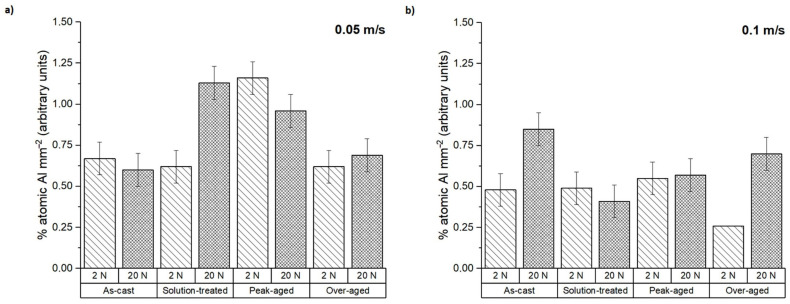
The atomic percentage of aluminum on the worn surface at sliding speeds of (**a**) 0.05 m s^−1^ and (**b**) 0.1 m s^−1^.

Furthermore, the presence of aluminum on the pin surfaces after testing was measured from the EDS spectra (Figure 7). It is worth mentioning that the tested samples do not contain aluminum in their composition. For this reason, the presence of aluminum indicates a material transfer from the counterbody to the tested pins during the dry sliding wear test. At the lowest sliding speed, the Al percentage ranged from 0.50 to 1.20%; the material transfer was considerably higher for the peak-aged condition and the solution-treated sample with an applied load of 20 N. However, at 0.1 m s^−1^, the transferred aluminum decreased, reaching an average value of 0.60% in all conditions.

### 3.4. Wear Mechanisms

**Figure 8:**
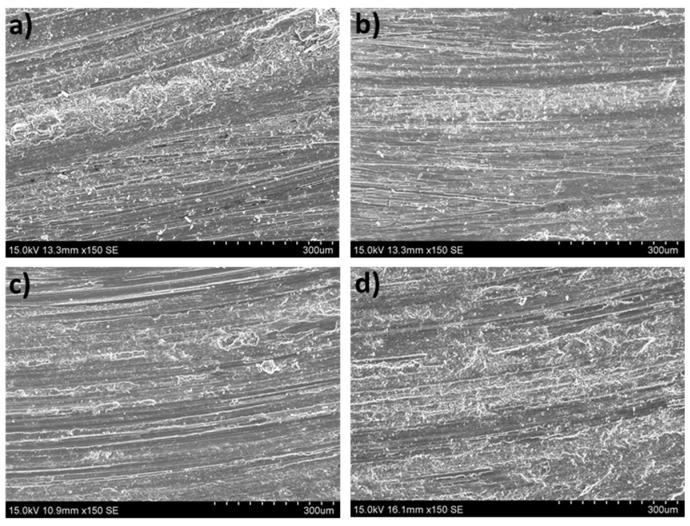
Worn tracks observed for the as-cast ZX30 alloy tested under the different wear conditions: (**a**) 2 N, 0.05 m s^−1^; (**b**) 20 N, 0.05 m s^−1^; (**c**) 2 N, 0.1 m s^−1^; (**d**) 20 N, 0.1 m s^−1^.

The worn surfaces and the debris formed were analyzed to determine the different wear mechanisms suffered by the tested samples. Figure 8 shows the worn surfaces of the as-cast ZX30 alloy samples tested under different load and scanning speed conditions. Abrasion and oxidation were the main wear mechanisms detected in this condition. The grooves observed parallel to the sliding direction indicate that abrasion took place [30]. These lines are caused by the presence of hard zones on the counterbody and cause the removal of small fragments of material and the formation of this type of pathway on the magnesium substrate. The measured O and Al contents (Figure 6 and Figure 7) indicate the presence of an oxidation process and material transfer from the counterbody to the pin surface. The presence of aluminum on the surface of the samples is observed as bright zones, and it is related to the formation of micro-joints between the counterbody and the sample [31]. The presence of oxides is evident and can be observed in the SEM micrographs as many small particles on the worn surface. The higher number of oxides observed on the worn track of samples tested at higher speeds suggests that, under these conditions, the aggressivity of the oxidative mechanism increased. This was due to the high activity of the magnesium and due to the temperature reached in the contact between the counterbody and the sample. These results are in accordance with the results obtained by other authors [18,32], where wear behaviors of different magnesium alloys at different wear conditions were obtained. They observed that the main wear mechanisms under such loads were oxidation and abrasion, regardless of the speed. Material pile-up is not observed under these conditions, which indicates that ductile ploughing does not occur.

**Figure 9:**
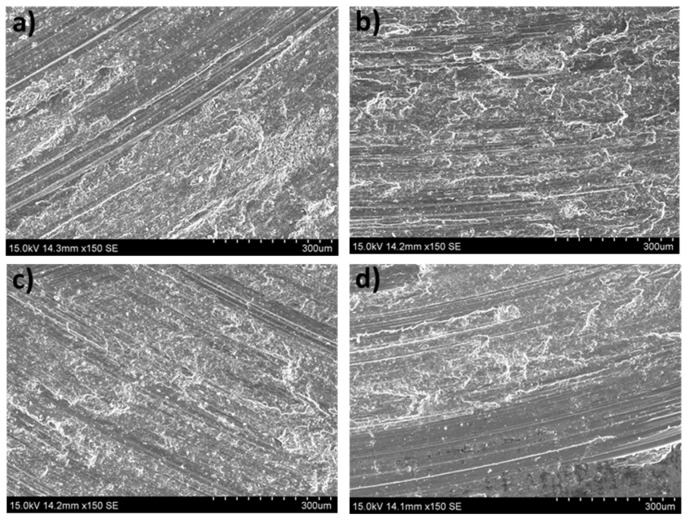
Worn tracks observed for the solution-treated ZX30 alloy under the different wear conditions: (**a**) 2 N, 0.05 m s^−1^; (**b**) 20 N, 0.05 m s^−1^; (**c**) 2 N, 0.1 m s^−1^; (**d**) 20 N, 0.1 m s^−1^.

According to Figure 9 and the results of the EDS analysis in Figure 6 and Figure 7, the wear mechanisms observed in the solution-treated ZX30 alloy samples correspond to abrasion and oxidation. This implies that they show wear rates like those found in the rest of the tested conditions. At the most energetic conditions (Figure 9d), deformation of the material at the surface was observed because of the load applied and the low hardness of the material [33]. It was also observed that, under these conditions, the worn surface showed zones with a high quantity of oxides and zones without oxides. This observation suggests that the oxidative layer formed during the wear process is not continuous, and it cannot act as a protective layer [34]. At low speeds and high loads, the presence of a high number of bright zones in comparison with the other samples suggests that the adhesion mechanism is the main wear mechanism under these conditions, which was also observed due to the high quantity of aluminum detected in the surface of the tested sample (Figure 7).

**Figure 10:**
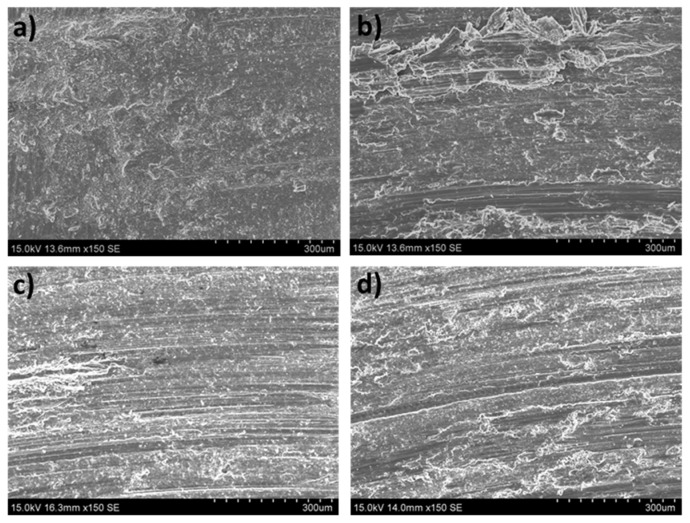
Worn tracks observed for the peak-aged ZX30 alloy under the different wear conditions: (**a**) 2 N, 0.05 m s^−1^; (**b**) 20 N, 0.05 m s^−1^; (**c**) 2 N, 0.1 m s^−1^; (**d**) 20 N, 0.1 m s^−1^.

From Figure 10 and considering the O and Al contents (Figure 6 and Figure 7), it can be deduced that the main wear mechanisms found in the peak-aged condition were also abrasion and oxidation. The peak-aged samples had an average hardness value of ~74 HV, which is higher than that of the AZ31 alloy (63.8 ± 2.6 HV) used as the counterbody. This hardness dissimilarity could have produced a local temperature increment in the contact area between the pin and the counterbody, leading to a rise in the wear rate, as observed under the most severe test conditions (Figure 2). However, the temperature reached seems to have been not high enough to induce the adequate oxidation rates required to form a protective oxide film, as other authors suggested [35].

**Figure 11:**
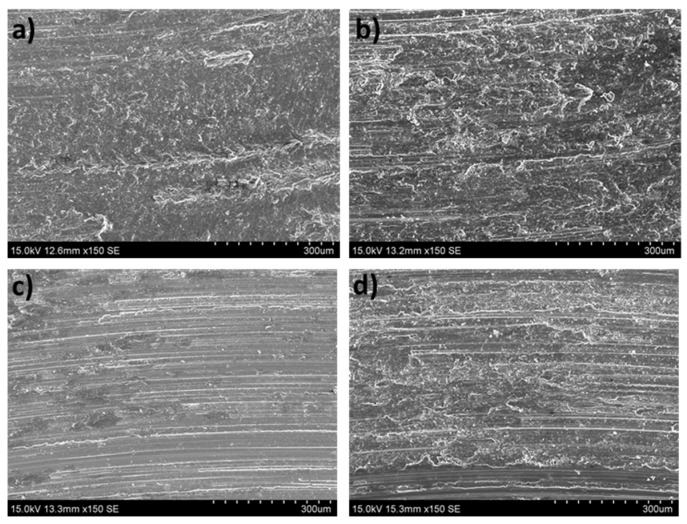
Worn tracks observed for over-aged ZX30 under the different wear conditions: (**a**) 2 N, 0.05 m s^−1^; (**b**) 20 N, 0.05 m s^−1^; (**c**) 2 N, 0.1 m s^−1^; (**d**) 20 N, 0.1 m s^−1^.

Figure 11 shows the worn surfaces of the over-aged ZX30 alloy samples. The main wear mechanisms correspond to abrasion and oxidation. Deformation of the material at the surface can be observed in most conditions. As in the rest of the studied ZX30 alloy samples, the over-aged samples presented a high amount of O on the surface (Figure 6), which indicates the tendency of this alloy to suffer an oxidative process. However, at low loads and high speeds, the number of oxides on the surface decreased, and the abrasive mechanism seems to have been the main mechanism, as the grooves formed on the surface of the sample are more pronounced.

**Figure 12:**
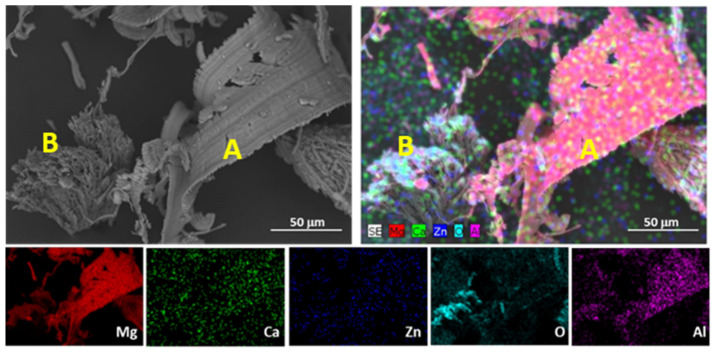
SEM micrograph and EDS analysis of the wear debris found in the over-aged ZX30 alloy samples at 20 N and 0.1 m s^−1^. A and B correspond to zones with different composition: A is richer in Al, and B is richer in O.

Figure 12 shows the wear debris generated because of the dry sliding test. It shows two different morphologies: the debris marked with A contained more Al, while the one marked with B had a higher O content. It is suggested that debris A, which has an elongated, curved, or spiral morphology, corresponds to the material removed from the counterbody during the wear test because the AZ31 counterbody contains Al in its composition. On the other hand, debris B refers to the oxide layer generated during the wear test. Debris B presents a sharp and irregular morphology resulting from the agglomeration of small, oxidized particles generated during the wear test. These particles could provide protection against wear to some extent.

**Figure 13:**
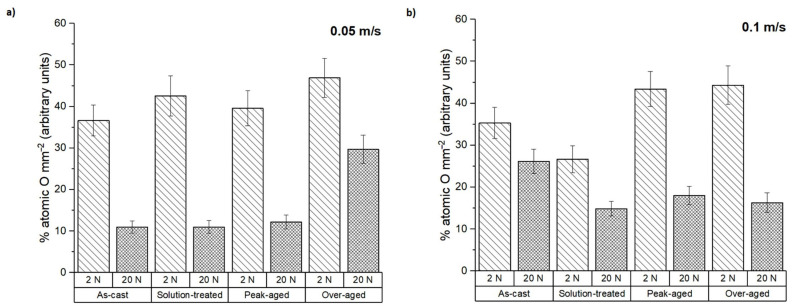
The atomic percentage of oxygen in the debris of the samples tested at (**a**) 0.05 m s^−1^ and (**b**) 0.1 m s^−1^.

**Figure 14:**
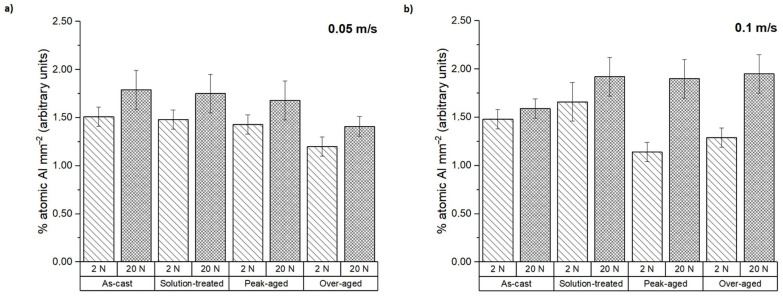
The atomic percentage of aluminum in the debris at sliding speeds of (**a**) 0.05 m s^−1^ and (**b**) 0.1 m s^−1^.

The compositional analysis of the debris generated under the different wear test conditions in terms of O and Al contents (atomic%) is represented in Figure 13 and Figure 14. For the ZX30 alloy, the measured O in all conditions was considerably lower at 20 N than at 2 N of applied load. This suggests that at lower load and sliding velocity, the oxidative phenomenon dominates. This result has been also observed in other alloys such as AM50B and AM60B [36,37]. As seen from Figure 14, the debris from all the samples presented a high Al content, being slightly superior at 20 N. This indicates that material transference from the AZ31 alloy (counterbody) to the tested sample took place during the wear test.

The heat treatments modify the microstructure and enhance the hardness of the ZX30 alloy, but this fact does not always lead to an improvement in the wear resistance. On the one hand, numerous larger precipitated phases can act as harder barriers that hinder some wear mechanisms, such as plastic deformation, in a similar way as ceramic reinforcements withhold load and extend the mild wear range of metal matrix composites [37]. On the other hand, these precipitated particles are more brittle and could break and detach from the surface, becoming harsh artifacts in the contact area and worsening the wear behavior by the abrasion mechanism. Furthermore, the oxidized layer was not protective in all the cases studied because a continuous layer was not always formed on the worn surface.

## 4. Conclusions

The following conclusions can be drawn:

- The ZX30 alloy showed a hardness value of ~61 HV in the as-cast condition, and its microstructure showed eutectic precipitated phases within the GB. After solution treatment, a small number of eutectic phases dissolved, and the redistribution of the undissolved ones in the GBs took place. Under the peak-aged condition, the hardness increased to ~74 HV due to the precipitation of Zn-rich phases and fine Ca_2_Mg_6_Zn_3_ particles. Despite the increase in hardness, the wear rate of the ZX30 alloy was not much affected by the heat treatments applied.
- The measured wear rates and friction coefficients were not significantly affected by the load applied, not showing strong differences between 2 N and 20 N. The friction coefficient was in the 0.30–0.40 range in all tested conditions.
- Abrasion and oxidation were the main wear mechanisms found in the ZX30 alloy under the different test conditions. The ZX30 tended to oxidize during wear testing.
- The composition and morphology of the wear debris after testing showed the coexistence of material from the ZX30 alloy (pin) and the AZ31 alloy (counterbody). Moreover, the transference of material from the counterbody to the pin was remarkable in most conditions tested, especially at lower sliding speeds.

## Figures and Tables

**Table 1:** Composition in wt.% of the ZX30 magnesium alloy.

	Composition in wt.%					
	Ca	Zn	Fe	Cu	Ni	Mg
ZX30	0.40	3.14	0.0120	0.0015	0.0008	Bal.

**Table 2:** Conditions tested with the aging time and Vickers microhardness.

Alloy	Sample	Aging Time (h)	HV0.1
ZX30	As-cast	-	~61
	Solution-treated	0	~63
	Peak-aged	4	~74
	Over-aged	24	~64

**Table 3:** Composition in wt.% of the AZ31 alloy used as a counterbody.

	Al	Ca	Cu	Fe	Mn	Ni	Si	Zn	Zr	TO *	Mg
AZ31	2.9	<0.005	<0.0005	0.005	0.17	0.0005	<0.005	0.96	<0.005	<0.3	Bal.

* TO: Total others.

## Data Availability

Data available on request.
